# Water-in-oil droplet-mediated method for detecting and isolating infectious bacteriophage particles via fluorescent staining

**DOI:** 10.3389/fmicb.2023.1282372

**Published:** 2023-12-06

**Authors:** Miu Hoshino, Yuri Ota, Tetsushi Suyama, Yuji Morishita, Satoshi Tsuneda, Naohiro Noda

**Affiliations:** ^1^Department of Computational Biology and Medical Sciences, Graduate School of Frontier Sciences, The University of Tokyo, Chiba, Japan; ^2^Biomedical Research Institute, National Institute of Advanced Industrial Science and Technology (AIST), Ibaraki, Japan; ^3^On-chip Biotechnologies Co., Ltd., Tokyo, Japan; ^4^Department of Life Science and Medical Bioscience, School of Advanced Science and Engineering, Waseda University, Tokyo, Japan

**Keywords:** bacteriophage, water-in-oil droplets, droplet isolation, plaque assay, bacteriophage screening

## Abstract

Bacteriophages are the most abundant entities on Earth. In contrast with the number of phages considered to be in existence, current phage isolation and screening methods lack throughput. Droplet microfluidic technology has been established as a platform for high-throughput screening of biological and biochemical components. In this study, we developed a proof-of-concept method for isolating phages using water-in-oil droplets (droplets) as individual chambers for phage propagation and co-cultivating T2 phage and their host cell *Escherichia coli* within droplets. Liquid cultivation of microbes will facilitate the use of microbes that cannot grow on or degrade agar as host cells, ultimately resulting in the acquisition of phages that infect less known bacterial cells. The compartmentalizing characteristic of droplets and the use of a fluorescent dye to stain phages simultaneously enabled the enumeration and isolation of viable phage particles. We successfully recultivated the phages after simultaneously segregating single phage particles into droplets and inoculating them with their host cells within droplets. By recovering individual droplets into 96-well plates, we were able to isolate phage clones derived from single phage particles. The success rate for phage recovery was 35.7%. This study lays the building foundations for techniques yet to be developed that will involve the isolation and rupturing of droplets and provides a robust method for phage enumeration and isolation.

## 1 Introduction

Bacteriophages (phages) are viruses that need to infect bacterial cells to propagate, which results in the lysis or slowed reproduction rate of host cells. This parasitic life cycle and the resulting deceleration in host cell growth coupled with the species-specificity establish phages as a next-generation antimicrobial agent with a focused area of action (Harada et al., 2018). As such, phages have begun to reattract attention for use as microbial control agents in fields such as medicine (Febvre et al., 2019; Rasmussen et al., 2020), agriculture (Zhou et al., 2013; Wang et al., 2019), and food safety (Goodridge et al., 1999a; Tian et al., 2022). With the progress of phage research, many interactions between phages and host cells were uncovered, such as the numerous phage resistance mechanisms of bacterial cells and phage-phage interactions (Erez et al., 2017; Borges et al., 2018). In this era of phage research, a high-throughput method of isolating phages from the environment would be of monumental value. Here, we propose a novel method to isolate viable phages from the environment, using model phages as a proof-of-concept.

Phages are thought to have been discovered independently by Twort (Twort, 1915) in 1915 and D’Herelle (D’Herelle, 2007) in 1917). Plaque assay has remained the gold standard for isolation and enumeration of phages since it was introduced over a century ago. This method and the phages obtained using this method have provided mankind with a fundamental understanding of molecular biology (Salmond and Fineran, 2015). However, the plaque assay has limitations such as the requirement of an even lawn of host cells. This limits the range of host cells to those that can grow on agar plates, preferably with limited mobility. In addition, various types of bacteria that have the ability to degrade agar (Chi et al., 2012), which may be problematic for their use as host cells in plaque assays. To overcome such limitations, we employed water-in-oil droplets (droplets) as a platform for segregating and cultivating phage particles.

**Figure 1:**
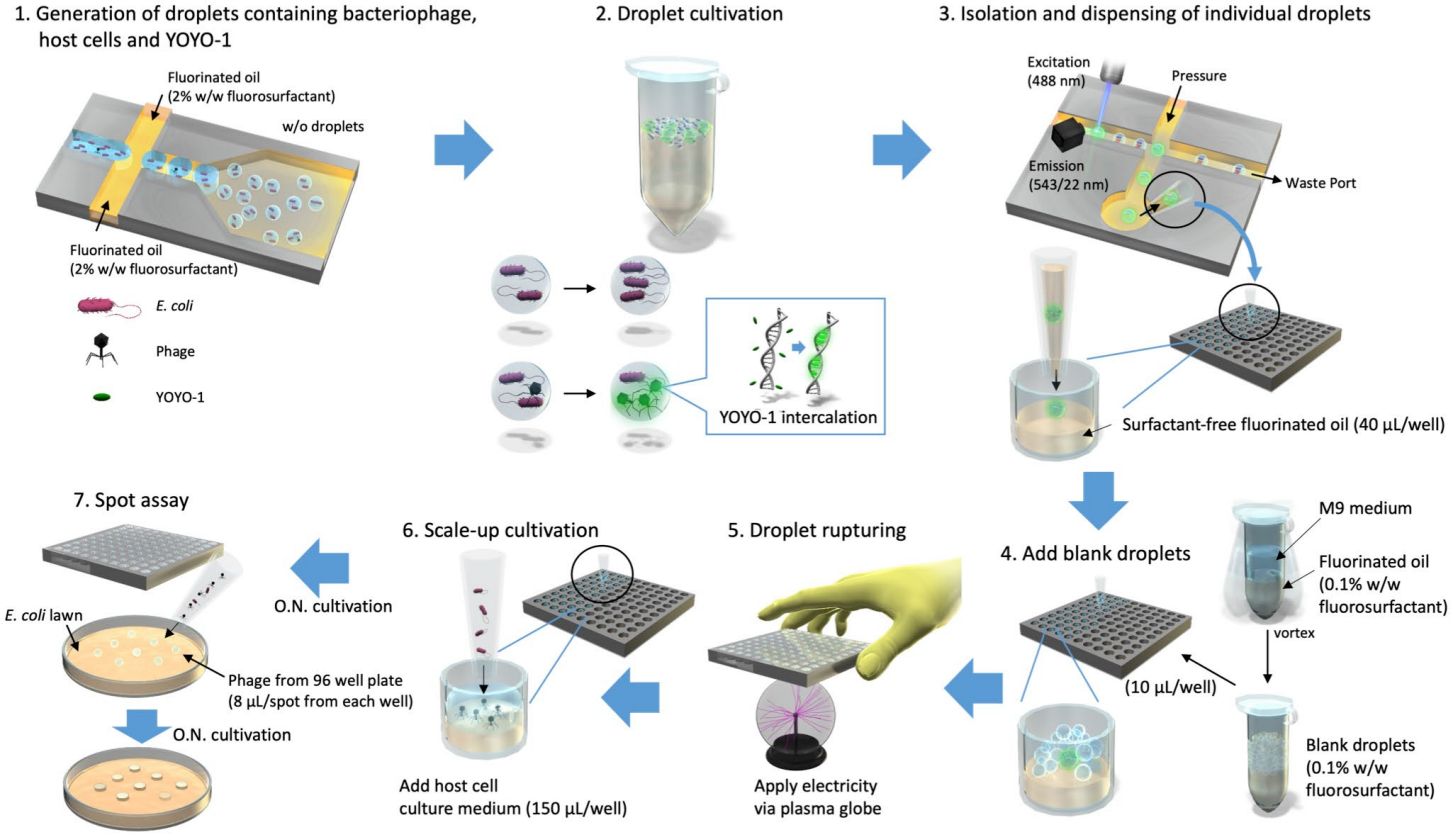
Isolation of droplets in which bacteriophage propagation occurred. Droplets containing T2 phage, *Escherichia coli*, and YOYO-1 were generated using On-chip Droplet Generator (Step1) and placed in a 30°C incubator to allow for bacteriophage infection to occur within droplets (Step 2). Following incubation, On-chip Droplet Selector was used to assess fluorescence intensities of individual droplets and isolate those which met a particular criterion regarding droplet size and fluorescence intensity. Droplets in this target range were isolated into individual wells of 96-well plates containing 40 μL/well of surfactant-free fluorinated oil along with an additional 10 μL of fluorinated oil (Step 3). Following isolation, droplets produced by emulsifying culture medium and fluorinated oil containing 0.1% (w/w) surfactant (referred to as “blank droplets”) were added to the 96-well plates (Step 4). These 96-well plates, containing a single target droplet, 10 μL of blank droplets, and 50 μL of fluorinated oil per well, were pressed onto an active plasma globe to induce a chain reaction of droplet rupturing (Step 5). This was conducted approximately 5 s per well. Subsequently, 150 μL of *E. coli* overnight culture was added to each well (Step 6) and incubated overnight (O.N.). The contents of each well were used to conduct a round of spot assay to monitor bacteriophage propagation within the well (Step 7).

In the last decade, droplet technology has emerged as a powerful high-throughput platform for screening micro-sized particles (Kaminski et al., 2016). Some uses for this technology in the field of microbiology include screening for microbial and fungal cells (Samlali et al., 2022) and enzymes (van Loo et al., 2019; Yanakieva et al., 2020), cultivation of rare or hard-to-culture microbes (Ota et al., 2019; Saito et al., 2021), and single-cell analyses (Terekhov et al., 2017; Pryszlak et al., 2022). This technology is typically used in combination with fluorescence to distinguish droplets containing target cells or enzymes from others. As such, technologies for differentiating droplets are also being developed, Woronoff et al. (2011), Ota et al. (2019), Nakamura et al. (2022) as are technologies to exchange the solution within droplets (Abate et al., 2010; Huang et al., 2021). Droplet-based technologies are based on the precondition that absolute compartmentalization of particles can be achieved upon generation of droplets. Similarly, our proposed method compartmentalizes phage particles into individual droplets upon generation, thereby effectively isolating phage particles. We employed a fluorescent dye to stain bacteriophage particles and differentiate droplets in which phage propagation occurred via fluorescence intensity. We selected YOYO-1 for this purpose, because its cell-impermeant characteristic prevents active staining of host cells, while its property as an intercalating dye actively promoted the staining of phage nucleic acid (Goodridge et al., 1999b), as shown in Figure 1 Step 2. In addition, YOYO-1 is a stable homo-dimer complex available for use which have numerous similar intercalators with differing excitation/emission wavelengths, such as TOTO-1 (Fürstenberg et al., 2006).

In this study, we demonstrated the ability to isolate viable phage particles via droplet technology. By co-encapsulating phages, host cells, and fluorescent dye within droplets, we could observe propagation of single phage particles in droplets through monitoring droplet fluorescence intensities. We successfully targeted and isolated individual droplets into 96-well plates for upscaled cultivation of isolated phages. Using YOYO-1, fluorescent staining of the general phage population in a sample is enabled without the use of any genetic recombination, making this a highly applicable method. This method is capable of yielding previously undiscovered phages for known host cells when applied to environmental samples. In addition, this method may enable tractable screening of phage even when using microbes that grow slowly or are difficult to handle on agar plates as host cells.

## 2 Materials and methods

### 2.1 Bacteriophage, host cell, and culture medium

*Escherichia coli* B (NBRC 13168) and T2 phage (NBRC 20002) were obtained from NITE Biological Resource Center (NBRC) culture Cells were cultured following NBRC’s recommended protocol in 802 culture medium and frozen at −80°C as glycerol stocks. Glycerol stocks of these cells grown in M9 culture medium were also created and stored at −80°C.

Prior to compartmentalization into droplets, *E. coli* cells were inoculated from the glycerol stocks (grown in M9 medium) into 2 mL of M9 culture broth and shaken overnight at 30°C for preculturing. Cell were then diluted (1,100) into fresh M9 medium and cultured for one more day. Resulting cells were washed 3 times and resuspended into fresh M9 medium at a concentration of 10^9^ cells/mL. This concentration was calculated based on optical density of the cell suspension at 600 nm (OD_600_), which was measured with GeneQuant 1,300 (Biochrom Ltd., United Kingdom). Phages were propagated by co-cultivation with their host cells in liquid 802 medium. Prior to co-cultivation with phage, *E. coli* host cells were inoculated into 2 mL of 802 medium from glycerol stocks and shaken overnight. This preculture was resuspended into two flasks containing 20 mL of 802 medium each and shaken for 1–2 h (30°C, 120 rpm), after which 200 μL of phage suspension was added to one of the flasks. Both flasks were shaken for an additional 3–4 h, and the contents of both flasks were combined. Following another 3–4 h of shaking, the contents of both flasks were centrifuged (4°C, 8,000 × *g*, 15 min) to remove remaining host cells. PEG 6000 (4 g) and sodium chloride (1.6 g) were added to the supernatant and vigorously vortexed. This mixture was cooled down by placement in a − 20°C freezer for 1 h prior to centrifugation (4°C, 10,000 × *g*, 60 min) to pellet phage. Supernatant was discarded, and the resulting phage pellet was resuspended in 6 mL of sodium magnesium (SM) buffer, which was subsequently washed three times with chloroform to remove debris. The resulting phage particles suspended in SM buffer were used as concentrated phage suspension.

### 2.2 Droplet generation and optical signal intensity assessment

Monodisperse droplets with diameters of approximately 30 μm were generated using 2D chip-800DG (On-chip Biotechnologies, Japan) attached to an On-chip Droplet Generator (On-chip Biotechnologies, Japan). After both the oil (200 μL/sample) and sample (40 μL/sample) phases were loaded into their respective reservoirs, both phases were simultaneously pressurized for 20 min to generate droplets (Figure 1 Step 1). The pressure applied to the oil phase was 27 kPa, and the pressure applied to the sample (aqueous) phase was 25 kPa. Following generation, droplets were collected into 0.6 mL sample tubes and incubated at 30°C (Figure 1 Step 2). Sample phases were prepared shortly before application to 2D chip-800 DG and droplet generation. The sample phase comprised 160 nM YOYO-1 (ThermoFisher Scientific, USA), T2 phage and *E. coli* in M9 medium, or YOYO-1 and *E. coli*. The oil phase used was HFE-7500 Novec Engineered fluid (3 M) with 008-FluoroSurfactant (Ran Biotechnologies, USA) mixed at 2% (w/w). On-chip Sort (On-chip Biotechnologies, Japan) and Chip-Z1001 (On-chip Biotechnologies, Japan) were used for measuring fluorescence intensities of droplet samples. For sheath oil, 0.1% (w/w) 008-FluoroSurfactant in HFE-7500 was used. A 488 nm wavelength laser was used as an excitation light source, and fluorescence intensities in the range of 543/22 nm and forward-scatter intensities were measured. For each droplet sample whose optical intensities were measured, optical intensities of approximately 20,000 droplets were measured and recorded to test YOYO-1 resolution. Similarly, approximately 40,000 droplets were measured and recorded to visualize changes in fluorescence intensity distribution of droplets generated with YOYO-1, phage, and host cells over time. In our experiments, we used On-chip Droplet Generator to generate approximately 1.0 × 10^6^ droplets in 20 min, and On-chip Sort to measure droplet fluorescence intensities at a rate of approximately 180 droplets/s.

### 2.3 Isolation of individual droplets

Fluorescence intensities of a few thousand droplets were measured using On-chip Droplet Selector (On-chip Biotechnologies, Japan) to produce plots showing the distributions of fluorescence intensity. Then fluorescence intensity boundaries of target droplets were chosen based on the graphs obtained from the measurements. For fluorescence intensity measurement, droplets were loaded into the sample port of 2D Chip-SD1000 (On-chip Biotechnologies, Japan), which was then set into the On-chip Droplet Selector. After setting the fluorescence intensity boundaries, fluorescence intensities of droplets were measured once more, and those with fluorescence intensities within the chosen threshold were sorted and isolated (Figure 1 Step 3). On-chip Droplet Selector works by pushing out droplets within the chosen threshold into a chamber for isolation and can isolate up to 96 droplets in 10 min. The isolated droplets are then automatically dispensed into a 96-well plate. Single droplet isolation was conducted into individual wells of 96-well plates pre-filled with 40 μL of HFE-7500. A 488 nm wavelength laser was used as an excitation light source, and fluorescence intensities in the range of 543/22 nm and forward-scatter intensities were measured.

### 2.4 Optical intensity signal analyses and data visualization

Fluorescence intensities of droplets were analyzed by importing the data of fluorescence intensities and forward-scatter intensities of droplet samples as csv files into Rstudio (ver. 4.1.0). Packages tidyverse (ver. 1.3.1), ggplot2 (ver. 3.3.5), ggridges (ver. 0.5.3), and ggExtra (ver. 0.9) were used for data manipulation and visualization. Forward-scatter intensities were used to filter out the measurements of droplets with abnormal sizes (see Supplementary Tables S1–S3 for details). To account for variations in forward-scatter intensity means across different time points, measurements of droplets with forward-scatter intensities deviating by more than one standard deviation from the mean intensity of their corresponding time point were removed.

### 2.5 Image analyses

Microscopic photos of droplet samples were taken with a Nikon Ti2 Eclipse microscope (Nikon, Japan) and NIS-Elements Basic Research software ver. 5.20.00 (Nikon). The photos were imported into WinRoof 2018 software ver. 4.8.0 (Mitani Corporation, Japan) to analyze the diameters of droplets. These measurements were used to calculate the volumes of droplets as required.

### 2.6 Droplet rupturing using static electricity

Equal volumes of M9 medium and HFE-7500 with 0.1% (w/w) 008-FluoroSurfactant were added to a 15-mL Falcon tube, or a 2-mL tube and emulsified by thorough mixing with a vortex. We termed the resulting emulsion “blank droplets.” Blank droplets were added to 96-well plates following droplet isolation, 10 μL/well, as shown in step four of Figure 1. As emulsions are prone to breaking when placed in an electric field (Karbaschi et al., 2017; Mizoguchi and Muto, 2019), an active plasma globe was used to generate an electric field while rupturing the droplets. To rupture isolated droplets, the bottom of the 96-well plate was pressed onto an active plasma globe to trigger a chain reaction of droplet rupturing using the added blank droplets (Figure 1 Step 5). During this step, the plate was slowly repositioned to allow all wells to be evenly stimulated by the plasma globe.

### 2.7 Digitization of phage existence within isolated droplets via spot assay

Overnight culture suspension of *E. coli* was added to the droplet solution (Figure 1 Step 6), which was obtained via isolation and rupturing in 96-well plates (150 μL/well). This was then incubated at 30°C overnight to promote further propagation of phages. To assess the existence of phage within the solution, spot assays were conducted using an agar plate made of 802 medium and a lawn of *E. coli*. This lawn was created by mixing 100 μL of *E. coli* suspension into molten 802 medium (0.7% w/v Lonza SeaPlaque Agarose) and pouring the entire mixture onto the original plate. After the lawn solidified, 8 μL of the solution was taken from each well of the 96-well plate containing isolated droplets and gently spotted on the lawn (Figure 1 Step 7). Plates were inverted and incubated overnight after the spots dried. Wells were counted as phage-positive if the solution used for spotting resulted in a difference on the *E. coli* lawn after incubation.

### 2.8 Plaque assay using phages within droplets

A fixed volume (8 μL) of droplets containing phage and *E. coli* was taken into 0.6-mL tubes containing 50 μL of HFE-7500 and 45 μL of M9 medium and vortexed. The sides of the tube containing this mixture were pressed onto a plasma globe for rupturing by slowly rolling it around to stimulate the emulsion as evenly as possible. The resulting sample was serially diluted with M9 medium to be used in plaque assays. Plaque assays were done in triplicate for each dilution, and one dilution for each timepoint was selected to calculate the average titer. The plaque assays were conducted using a modified version of the protocol by Kropinski et al. (2009).

## 3 Results

We developed a droplet-based approach for isolating bacteriophage particles which we evaluated using a spot assay. As shown in Figure 1, this method starts by co-encapsulating T2 phage particles with their host cell *E. coli* into micro-sized droplets with YOYO-1 iodide (Step 1). These droplet samples are placed in an incubator to allow for the occurrence of in-droplet phage infection and propagation (Step 2). When phage infection and subsequent propagation occurs, YOYO-1 intercalates into DNA of progeny phage, thereby distinguishing droplets with phages from those without by fluorescence intensity. The droplets in which phage propagation occurred are then isolated into individual wells of 96-well plates pre-filled with 40 μL/well of HFE-7500 (Step 3). The isolated droplets are then ruptured and subjected to scale-up cultivation. The first of these steps involves the creation of “blank droplets” by emulsifying culture medium and fluorinated oil containing 0.1% (w/w) fluorosurfactant. These “blank droplets” are added to 96-well plates (10 μL/well), into which droplet isolation was conducted (Step 4). Following the addition of “blank droplets,” the 96-well plate is pressed on the top of an active plasma globe (Step 5). This electrical stimulation is used to induce a chain reaction of droplet rupturing. Host cell culture is added to each well (150 μL/well) for scale-up cultivation of phage, which were within isolated droplets (Step 6). To evaluate the success rate of our method, we checked whether the whole process, from the generation of droplets to the isolation and scale-up cultivation of phages, yielded phage in 96-well plates. This was done by conducting a spot test with the sample phase of the 96-well plate (Step 7).

### 3.1 Fluorescence intensities of droplets containing bacteriophage suspension and YOYO-1 iodide

**Figure 2:**
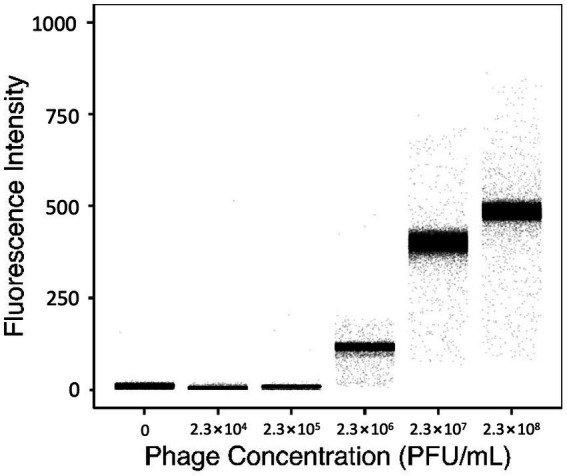
Fluorescence intensities of droplets containing 160 nM YOYO-1 and various concentrations of phage suspension. The average number of PFU within individual droplets for each sample shown were 0, 0.08, 0.8, 8, 80, and 800 PFU/droplet respectively, from the leftmost sample.

To evaluate the resolution of YOYO-1 iodide, the fluorescence intensities of droplets generated with 160 nM YOYO-1 and bacteriophage suspension at various final concentrations were measured. Fluorescence intensities of droplets generated with bacteriophage suspensions at final concentrations of 0, 2.3 × 10^4^, 2.3 × 10^5^, 2.3 × 10^6^, 2.3 × 10^7^, and 2.3 × 10^8^ plaque forming units (PFU) per mL are shown in Figure 2. Fluorescence intensities of droplet samples increased concurrently with phage concentration, with medians of 11.0, 5.0, 9.0, 116, 400, and 485 starting from the sample containing the lowest concentration of phage suspension. The sample without phage, which contained deionized water instead of phage suspension, had a slightly higher fluorescence intensity medium compared to the other samples containing low concentrations of phage.

The number of phage particles within individual droplets is given as “λ_phg_” which can be calculated from the concentration of phage suspension (Supplementary Equation S1). Here, the λ_phg_ depends on the phage concentration of the solution used to generate droplets. Samples with higher λ_phg_ generally had a broader distribution of fluorescence intensity, whereas samples with lower λ_phg_ had narrower distributions. The rightmost sample in Figure 2 had a λ_phg_ of approximately 800. This was calculated based on results obtained from a standard plaque assay protocol using the same phage suspension used to create the droplet sample. Fluorescence intensities of the sample generated with 2.3 × 10^5^ PFU/mL and the sample generated with 2.3 × 10^4^ PFU/mL were similar. However, fluorescence intensities of these samples and the sample generated with 2.3 × 10^6^ PFU/mL were different. These results show that droplets generated with 160 nM YOYO-1 iodide have fluorescence intensities dependent on the concentration of phage within droplets. In other words, the fluorescence intensity of a droplet, in comparison with other droplets, can be used to determine its phage content in comparison to other droplets.

### 3.2 Transition of droplet fluorescence intensity and titer

**Figure 3:**
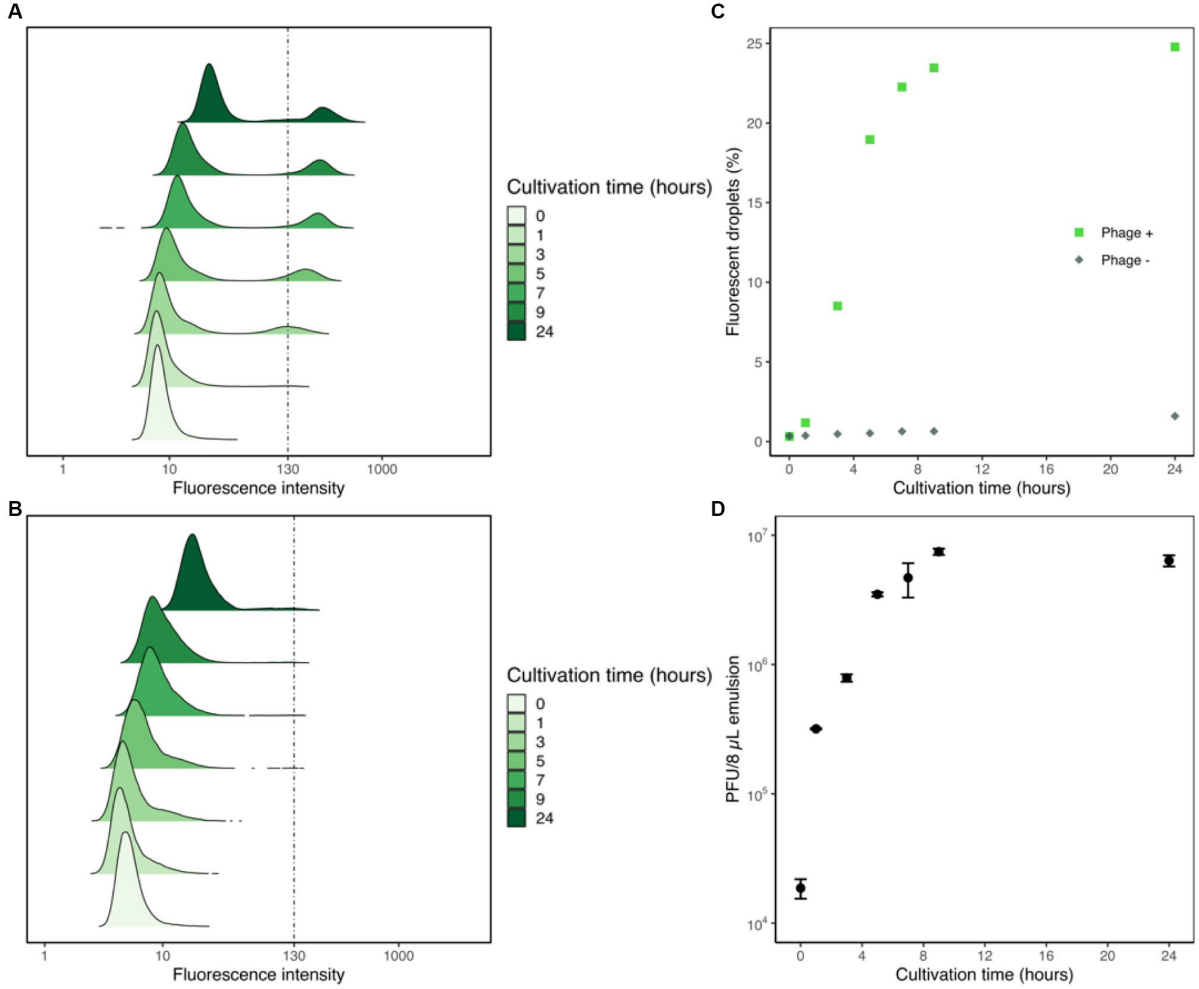
Flow cytometric analyses of droplet samples with and without phage. Fluorescence intensities of droplet samples containing phage, *E. coli*, and YOYO-1 **(A)** and samples with *E. coli* and YOYO-1 **(B)** were measured to create density ridgeline plots. These measurements were used to plot the percentages of droplets with fluorescence intensities higher than 130 **(C)**. Concurrent with fluorescence intensity measurements, a fixed volume (8 μL) of droplets was sampled and ruptured from the phage containing droplet samples to perform a titer check via plaque assay **(D)**.

To assess the correlation between droplet fluorescence intensities and the corresponding phage content, we tracked the changes in fluorescence intensities of droplets generated with YOYO-1, phage, and host cells, and measured the titer of phage suspension within the droplets via plaque assay. Droplet samples containing T2 phage, *E. coli*, and YOYO-1 were generated and incubated at 30°C, as were droplets containing only *E. coli* and YOYO-1. For both samples, YOYO-1 was used at a final concentration of 160 nM. At the 0, 1, 3, 5, 7, 9, and 24-h time points, fluorescence intensities of approximately 40,000 droplets were measured. These measurements were used to plot the transformation of density distribution in droplet fluorescence intensity over time (Figures 3A,B). In the sample containing phage (Figure 3A), a subgroup of droplets with a strong fluorescence intensity could be observed. This subgroup was not observed in the sample generated without phage (Figure 3B). This subgroup, which appeared during the first hour of incubation, increased in both proportion and fluorescence intensity as incubation progressed. During the first 9 h of incubation, the two groups of droplets with high and low fluorescence intensities in Figure 3A were clearly distinguishable from each other. Conversely, while the two groups of droplets could be divided into fluorescent and non-fluorescent droplets even after 24 h of incubation, a trace proportion of measurements fell mid-way between the two groups. This group of droplets with medium fluorescence intensities could also be clearly seen in the sample generated without phage, incubated for 24 h. Similarly, droplets with medium fluorescence intensities were also observed in the samples generated without phage, incubated for 7 and 9 h. The percentage of droplets with fluorescence intensities stronger than 130 were calculated and plotted as Figure 3C. As less than 0.5% of droplets without phage measured at the 5-h time point had fluorescence intensities higher than 130, this was chosen as the fluorescence intensity differentiating fluorescent droplets and non-fluorescent droplets. In the sample containing phage, a sharp increase in the percentage of fluorescent droplets could be observed until 5 h of incubation. After 5 h, a sharp decrease in the differential of fluorescent droplets to cultivation time was observed. In contrast with the phage-containing sample, the proportion of fluorescent droplets in the sample prepared without phage remained relatively low throughout the whole 24 h.

Alongside the measurements of droplet fluorescence intensities, the phage titers within the droplets were also measured. To do this, 8 μL of emulsion from the droplet sample generate with phage was sampled to use in plaque assays at each timepoint. The results of these plaque assay are plotted in Figure 3D. The initial hour of incubation resulted in an over 17-fold increase in the phage titer within droplets. After the first hour, phage titer within 8 μL of emulsion continued to increase steadily, albeit at a lowered rate, until 9 h of incubation. Contrary to the surge in phage population during the first few hours of incubation, phage titer within 8 μL of emulsion measured after 9 h and 24 h were comparable. Together these results show that phage infection and propagation are indeed occurring within droplets, and that this increase in phage population causes droplet fluorescence intensity to increase as well.

### 3.3 Visualization of temporal changes within droplets

**Figure 4:**
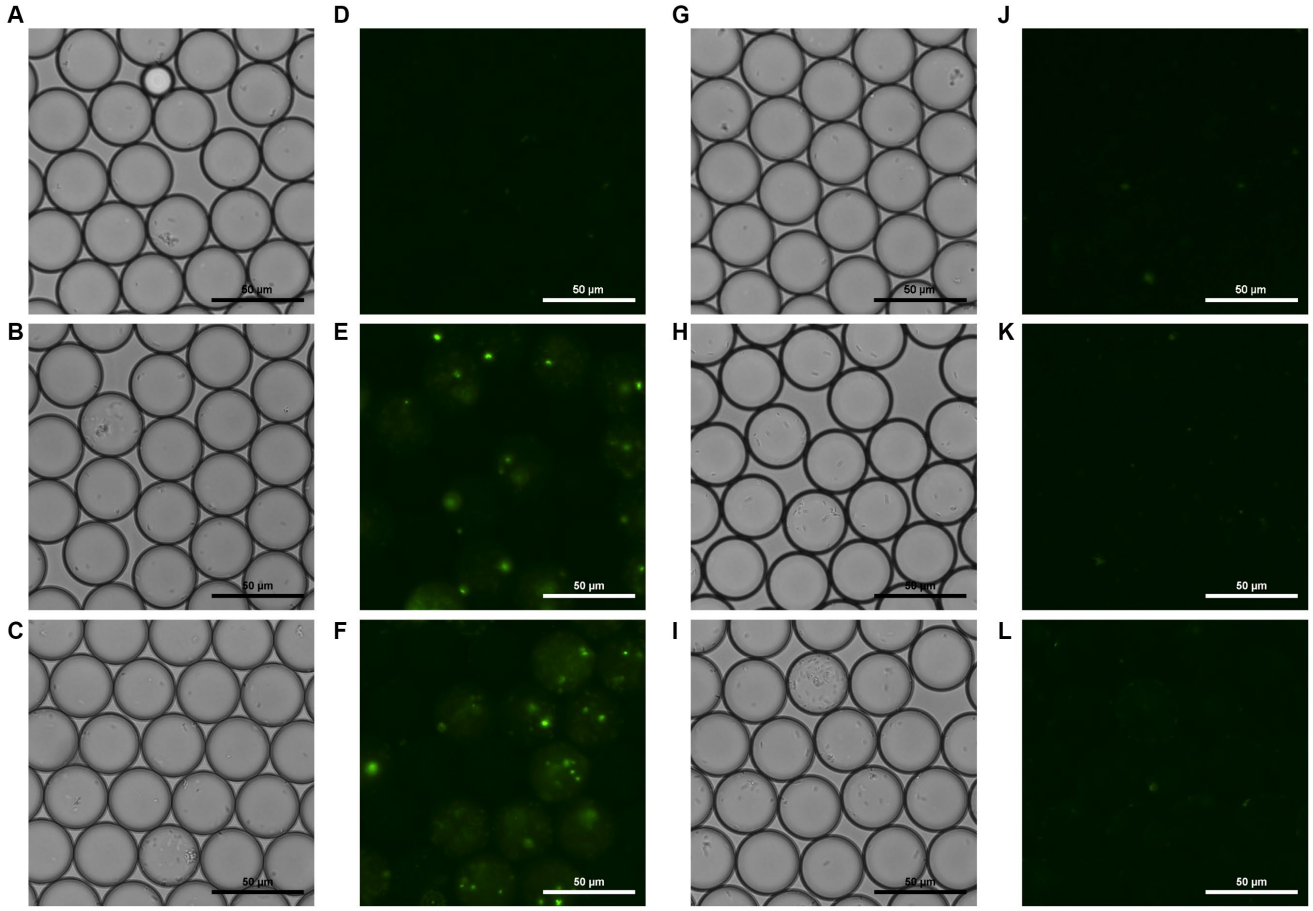
Bright field and fluorescent field microscopic photos of droplets containing phage, *E. coli*, and YOYO-1 **(A–F)**, and droplets containing *E. coli* and YOYO-1 **(G–L)**. **(A,D,G,J)**, were taken right after droplet generation. **(B,E,H,K)**, were taken after 3 h of static incubation at 30°C, and **(C,F,I,L)** were taken after approximately 5 h of static incubation at 30°C.

To document visible temporal changes in droplets, we captured sequential photographs of droplet samples. Microscopic photos of both the droplets generated with (Figure 4 left side) and without phage (Figure 4 right side) were taken. These photos were taken at 0, 3, and 5 h after incubation, following generation. Bright field and fluorescent field microscopic photos of droplets were taken. Comparison of these images revealed a difference in overall fluorescence intensity, which amplified over time. Figure 4F shows bright green spots as well as hazy circular droplets around them, which were not visible in 4 L. Fluorescent droplets can be observed in Figure 4E, demonstrating that fluorescence intensifies enough to enable differentiation via microscopic observation by the third hour of incubation. Furthermore, the number of *E. coli* cells that could be observed microscopically within droplets varied between the two samples, and this difference was seen as intensifying over time.

### 3.4 Isolation of individual droplets and upscaled cultivation of phage

To test the effectiveness of our phage isolation method, we employed our model to obtain phages from individual droplets. To do this, droplets containing T2 phage, *E. coli,* and YOYO-1 were generated and incubated for 5 h. After 5 h, On-chip Droplet Selector was used to dispense droplets within the target range of fluorescence intensity and forward scatter intensities. Two target ranges were chosen here, with one being droplets with high fluorescence intensities, and the other being droplets with low fluorescence intensities. For each target range, droplet isolation into 96-well plates was conducted into at least 184 wells. Following isolation, droplets were ruptured using static electricity. Subsequently, fresh *E. coli* cultured in M9 medium was added to the wells and incubated overnight to promote further propagation of phage. These upscaled culture solutions in 96-well plates were used to conduct spot assay to determine the existence of phage. Spot assay results of wells containing droplets with high fluorescence intensities showed that 82/230 (35.7%) wells were phage-positive. In contrast, for wells containing droplets with low fluorescence intensities, 0/184 (0%) were phage-positive. Supplementary Figure S1 displays spot assay results from 32 of wells containing droplets with high and low fluorescence intensities. These results show that droplets with low fluorescence intensities did not contain phage, demonstrating that droplets containing viable phage will have a high fluorescence intensity.

## 4 Discussion

There have been studies reporting the rapid enumeration of phage using droplets, both by means of propagation and without. These propagation-based studies include the use of LacZ phage (Tjhung et al., 2014), and indirect observation of phage via analysis of light scattering (Yu et al., 2014). Phage enumeration without propagation was conducted by droplet digital PCR of phage genome sequences (Morella et al., 2018). In our method we used YOYO-1 to fluorescently stain progeny phage, and successfully differentiated droplets in which phage propagation occurred. Isolation and rupturing of fluorescent droplets, followed by upscaled recultivation of phage resulted in successful recovery of phage from individual droplets. To the best of our knowledge, this is the first study to propose an approach to isolate target droplets and test the success rate of the isolation and recultivation of single phage particles.

### 4.1 Probability calculations using the Poisson distribution

The number of particles encapsulated into droplets follow the Poisson distribution, meaning that the λ_phg_ is the mean number of phage particles in a droplet sample, and does not indicate the number of particles in each droplet. This difference in the number of phage particles confined in each droplet can be seen in Figure 2, where the droplet samples generated with a higher concentration of phage suspension had broader distributions of fluorescence intensity.

The probability of a droplet containing a specific number of phage particles can be calculated from the λ_phg_ value, and the numerical range of phage particles enclosed within individual droplets increases as the λ_phg_ increases. This increase in numerical range likely resulted in a broadening of fluorescence intensity range, as can be observed in the droplet samples generated with 2.3 × 10^7^ PFU/mL and 2.3 × 10^8^ PFU/mL phage suspension. However, when λ_phg_ was sufficiently small, as was the case with the droplet sample containing 2.3 × 10^4^ PFU/mL phage suspension, the vast majority of droplets generated were likely devoid of phage particles. With the λ_phg_ of this sample estimated to be 0.08, 92.3% of droplets in this sample are expected to lack phages following the Poisson distribution, and 96.0% of phage-containing droplets are expected to contain a single phage particle. Thus, we can conclude that in samples with a sufficiently small λ_phg_, droplets likely contain no, or a very small number of phage particles, resulting in a narrow range of fluorescence intensity. This pattern can be observed in the droplet sample containing 2.3 × 10^5^ PFU/mL phage suspension, which had a λ_phg_ of 0.8, and similarly had a narrow distribution of droplet fluorescence intensity.

Contrary to the similarities between the samples whose λ_phg_ were 0.08 and 0.8, the sample with λ_phg_ of 8.0 (sample containing 2.3 × 10^6^ PFU/mL phage suspension) had a distinctly different distribution of droplet fluorescence intensity. This difference in fluorescence intensity implies that the resolution limit of this method ranges between 1 and 8 phage particles.

There are two ways to calculate λ_phg._ The first way is to calculate λ_phg._ Based on droplet size and titer of phage solution and the λ_phg_ of this sample calculated from phage titer was 0.4. The second way is to calculate it from the fraction of non-fluorescent droplets using the Poisson distribution. From the results in section 4.4, we concluded that droplets containing viable phage will have elevated fluorescence intensities after cultivation. In other words, droplets with low fluorescence intensities even after cultivation do not contain viable phage. This indicates that the fraction of non-fluorescent droplets in the sample generated with phage is the fraction of droplets devoid of phage, and the Poisson distribution can be used to calculate the λ_phg_ of the droplet sample before cultivation (Supplementary Equation S2). Using the fractions of non-fluorescent droplets measured after 5 h of cultivation, the λ_phg_ value of the droplet sample generated with phage in Figure 3 was approximately 0.2.

### 4.2 Phage-host interaction within droplets

Given that YOYO-1 is cell-impermeant, live *E. coli* cells are not expected to exhibit a strong fluorescence. Therefore, the increase in fluorescence intensity observed in droplets containing YOYO-1, phage and host cells can be attributed primarily to the intercalation of YOYO-1 into the nucleic acid of phage. Together with the observation that the subgroups of droplets with strong fluorescence intensities observed in Figure 3A appeared only after cultivation, we can deduce that phage infection and propagation occurred in these droplets, and that these droplets contain a substantial amount of phage. This phage propagation resulted in an increase in phage nucleic acid, into which YOYO-1 intercalation occurred, consequentially raising the fluorescence intensity of the droplet. A sharp increase in percentage of fluorescent droplets seen until 5 h of cultivation can be observed (Figure 3A), which continued to increase at a slower pace after this time point. This slowdown in the increase of fluorescent droplet percentage implies that the phage infection and release of progeny phage occurred at least once within the first 5 h of cultivation in most droplets that contained phage. Notably, approximately 5 h of incubation produced only miniscule plaques in plaque assay experiments conducted using T2 phage and *E. coli* B (Supplementary Figure S2), while it was enough time for phages to propagate enough within droplets to allow for clear differentiation from non-fluorescent droplets.

When the changes in percentage of fluorescent droplets are compared to the changes of phage titer within droplets, the first hour of cultivation resulted in an intense increase of phage titer, but not in the percentage of fluorescent droplets. From these results, it can be inferred that the number of phages after 1 h of cultivation was not sufficient to significantly increase fluorescence intensities. Comparison of these two parameters between the 9 and 24-h time points reveal a small increase in the ratio of fluorescent droplets, and a small decrease in phage titer. The lack of increase in phage titer indicates additional infection and propagation of phage did not occur within this 15-h time period, which contradicts the slight increase in proportion of fluorescent droplets. The increase in fluorescence may be attributed to host cell DNA and cell debris derived from lysed cells. Possible factors constraining phage population growth may include the host cells’ developing a resistance to phage, and the obliteration of the host cell population confined with the phage.

The aforementioned contradiction between fluorescent droplet percentages and phage titer indicates that other factors affected fluorescence intensities. These other factors may include the deterioration of *E. coli* cells during the stationary growth phase. Starvation of *E. coli* cells result in a loss of membrane integrity, which allows for nutrients to leak out of the cells (Schink et al., 2019). This weakness of cell membrane could allow for YOYO-1 to enter the cell, or nucleic acids to leak out, along with other cell debris resulting in elevated fluorescence not attributed to phage. This phenomenon could justify the presence of droplets with slightly elevated fluorescence intensities in both the sample generated with and without phage. We hypothesize that such droplets were generated with a higher-than-average number of *E. coli* cells by chance, resulting in accelerated depletion of nutrients, hence starvation.

### 4.3 Recovery of phages derived from single droplets

The results of droplet isolation and upscaled cultivation of phage in our research had a success rate of 35.7%, while previous studies on single droplet isolation and recultivation of *E. coli* inside reported a minimum of 80% recovery rate when upscaling on solid media (Weber et al., 2022). Although the latter study produced high recovery rates on solid medium, the recovery rates when liquid medium was used was 31%, which was similar to our phage recovery results. Note that our results are the recovery rate of phage, which require the additional steps of phage propagation following droplet isolation and rupturing. Some reasons for these relatively low recovery rates may include the loss of the target droplet, and the fluorescence of the droplet being unrelated to phage propagation, as mentioned in section 5.2. Target droplets may be lost due to the On-chip Droplet Selector accidentally isolating a droplet different than the target one, the droplet rupturing being unsuccessful, which prevents the upscaling of phages, or the target droplet becoming lost due to its small size. Individual droplets have a volume of approximately 18.8 pL which is very small relative to the wells of a 96-well plate and could be easily lost during steps subsequent to isolation, for example by adsorption onto pipette tips. These steps include but are not limited to the additions of blank droplets (Figure 1 Step 4) and culture medium (Figure 1 Step 6).

Despite the chance of droplet loss during the isolation and upscaled cultivation steps, we believe our proposed method’s throughput and ability to compartmentalize particles can compensate for this. Our method requires only a small phage concentration upon droplet generation to enable the isolation of individual phage particles and simultaneous differentiation of viable phage from non-viable ones. This method is capable of determining phage concentration of the original phage solution post-segregation and cultivation with host cells and can be applied to screen viable phage particles from environmental samples. The compartmentalizing characteristic of this method eliminates the need to conduct numerous rounds of plaque assay to completely isolate phage particles, as long as the initial phage concentration is set sufficiently low. This can drastically shorten the process of phage screening, which may be useful in scenes such as phage therapy. Since phages isolated using our method require further upscaling and purification for use in scenes such as phage therapy, the YOYO-1 that was within the single isolated droplet should become diluted enough to lose relevance.

In conclusion, we developed a method to simultaneously enumerate and isolate viable phage particles by co-cultivation inside droplets. This method of phage detection is expected to enable easy and high-throughput screening of lytic phages that infect a particular host cell. In addition, the liquid-based cultivation of this method may enable the use of bacteria with slow growth and growth restricted by agar as host cells. By establishing a method to isolate, rupture, and recultivate target droplets into liquid medium, we have successfully laid the foundations for future droplet-based high-throughput bacteriophage screening experiments.

## Data availability statement

The raw data supporting the conclusions of this article will be made available by the authors, without undue reservation.

## Author contributions

MH: Conceptualization, Data curation, Formal analysis, Investigation, Methodology, Software, Validation, Visualization, Writing – original draft, Writing – review & editing. YO: Conceptualization, Data curation, Investigation, Methodology, Writing – review & editing. TS: Conceptualization, Data curation, Methodology, Writing – review & editing. YM: Conceptualization, Data curation, Investigation, Methodology, Writing – review & editing. ST: Conceptualization, Data curation, Project administration, Supervision, Writing – review & editing. NN: Conceptualization, Data curation, Funding acquisition, Methodology, Project administration, Resources, Supervision, Writing – review & editing.
