# Supplementary material for: Water-in-oil droplet-mediated method for detecting and isolating infectious bacteriophage particles via fluorescent staining
Source: Front Microbiol. 2023 Dec 6;14:1282372. doi: 10.3389/fmicb.2023.1282372 (PMC10731258; doi:10.3389/fmicb.2023.1282372)
Supplement: Supplementary file 1 [file Data_Sheet_1.PDF]

Supplementary Information

Table S1. Number of droplets assessed and plotted to create Figure 2.

Phage concentration (PFU/mL)	Total number of droplets assessed	Number of droplet data excluded	Number of data used to create figure
2.3×10^8	20082	585	19497
2.3×10^7	20048	6522	13526
2.3×10^6	20082	584	19498
2.3×10^5	20045	810	19235
2.3×10^4	20104	1117	18987
0	20177	1116	19061

Table S2. Number of droplets assessed and plotted to create Figure 3a.

Time Point (h)	Total number of droplets assessed	Number of droplet data excluded	Number of data used to create figure
0	40044	1528	38516
1	40058	1832	38226
3	40140	2291	37849
5	40089	3065	37024
7	40070	4073	35997
9	40177	2549	37257
24	40136	2466	37670

Table S3. Number of droplets assessed and plotted to create Figure 3b.

Time Point (h)	Total number of droplets assessed	Number of droplet data excluded	Number of data used to create figure
0	40051	2729	37124
1	40123	4082	36041
3	40132	5489	34643
5	40082	2574	37508
7	40090	2435	37655
9	40017	2389	37628
24	40053	2689	37364

Supplementary Information

Equation S1. Calculation of the λ_{phg} using the titer of the original phage solution.

Titer of original phage solution : X PFU/mL
Average volume of individual droplets upon generation, calculated from droplet diameters : Y pL
Average number of phage particles in a droplet (λ_{phg}):

$$\lambda_{phg} = \frac{X\ (PFU/10^9 pL)}{Y\ (pL)}$$

Equation S2. Calculation of particle concentration (λ) using Poisson Distribution.

Poisson distribution formula:

$$P_{(m)} = \frac{\lambda^m e^{-\lambda}}{m!} \tag{1}$$

$P_{(m)}$: Probability of a droplet having *m* particles
λ: average number of phage particles in a droplet
Because droplets without phage (*m = 0*) are non-fluorescent, *m = 0* and the percentage of non-fluorescent droplets can be used to gauge the initial λ.

$$P_{(0)} = \frac{\lambda^0 e^{-\lambda}}{0!}$$
$$= e^{-\lambda} \tag{2}$$

$$lnP_{(0)} = ln\, e^{-\lambda}$$
$$= -\lambda \tag{3}$$

$$\boldsymbol{\lambda = -lnP_{(0)}} \tag{4}$$

Supplementary Information

a b

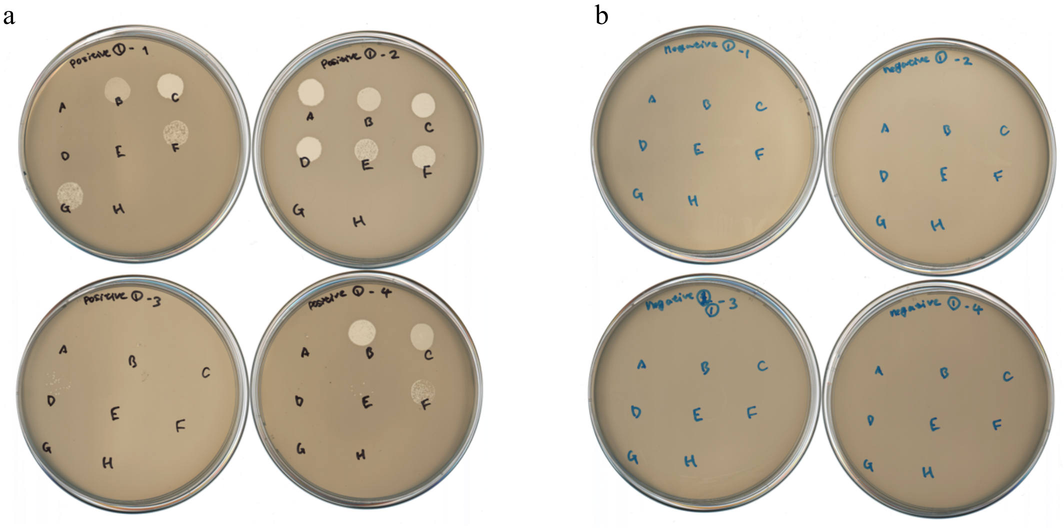

Figure S1. Plates after spot assay of culture medium derived from scale-up cultivation of isolated single droplets. Droplet samples containing T2 phage, *E. coli* and YOYO-1 were generated and incubated. After 5 h of incubation, droplets with high fluorescence intensities (S1a) and low fluorescence intensities (S1b) were isolated into 96-well plates, where steps for droplet rupturing and scale-up cultivation were performed. These culture media were scaled-up from individual droplets and used for spot assays. The letters in the photos depict the rows (A–H) of the 96-well plate from which the spot assays were conducted, and the numbers written on at the top depict the 96-well plate name (Positive①, Negative①) and columns (1–4). Numerous spots are observed in S1a, while no spots were observed in any of the plates shown in figure S1b, where spot assays using culture media scaled up from droplets with low fluorescence intensities were performed.

Supplementary Information

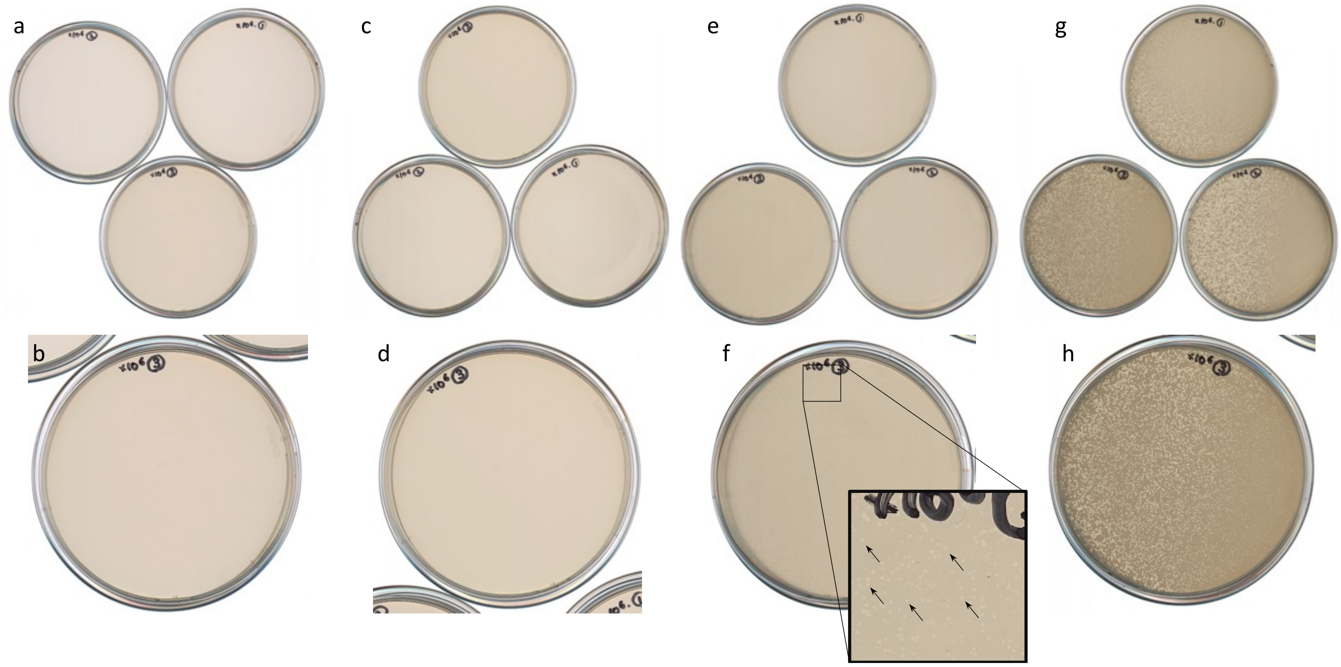

Figure S2. Surveillance of plaques over time.
The T2 phage and *E. coli* were mixed into a 802 soft agar solution to produce plaques. The plates onto which 3 mL each of soft agar was poured over normal 802 agar solution (n = 3) were incubated at 30 ºC, removed and photographed after approximately 1 (a, b), 3 (c, d), 5 (e, f), and 24 (g, h) h of plating. A slight darkening of the overall color of the plates was observed as the incubation time increased, due to host cell growth. Plaques started to appear after 5 h of incubation (indicated with arrows) and continued to become clearer with incubation time.
